# Mystical Myositis: A Case Series from Kalafong Provincial Tertiary Hospital, Pretoria, South Africa

**DOI:** 10.1155/2024/7410630

**Published:** 2024-08-05

**Authors:** Michael Myburgh

**Affiliations:** Department of Internal Medicine Kalafong Provincial Tertiary Hospital, PretoriaUniversity of Pretoria, South Africa

## Abstract

Idiopathic inflammatory myositis (IIM) is an expanding field in rheumatology as more myositis-specific antibodies (MSAs) and myositis-associated antibodies (MAAs) become available for testing. Clinical signs and specific clinical phenotypes are found in the MSA group, with as high as 70% of IIM patients having a positive myositis-specific antibody. Although IIM remains a heterogenous disease, assigning a phenotype to these patients will prove to be critical as we learn which cases require more aggressive therapy and what complications to search for as the disease progresses. The IIM patients for the last 5 years were reviewed and profiled using recently available myositis profile testing at our National Health Laboratory Services. Patients from our rheumatology clinic were categorized according to this antibody profile. Three cases diagnosed with dermatomyositis (DM) were selected for discussion in this article which include a patient with each of the following: anti-transcriptional intermediary factor 1-y (TIF1y) DM, anti-melanoma differentiation-associated protein 5 (MDA 5) DM, and anti-signal recognition particle (SRP) DM.

## 1. Introduction

Idiopathic inflammatory myopathies are a heterogenous group of disorders resulting in chronic inflammation of skeletal muscle tissue. Subclassification of IIM includes dermatomyositis, immune-mediated necrotizing myopathy, anti-synthetase syndrome, sporadic inclusion body myositis, polymyositis, overlap myositis, and juvenile dermatomyositis [1, 2].

Identification of these subtypes of IIM is important, and identification of the multiple subtypes within these domains with the help of particularly myositis-specific antibodies and clinical features may have important implications for the patient. For example, when dealing with an anti-MDA 5 DM patient who may go on to develop aggressive ulceration of the skin or even rapidly progressive interstitial lung disease (RPILD), early, effective therapy is essential [3]. A patient with anti-TIF1y DM has to be evaluated for a malignancy [1, 4]. A patient presenting with anti-NXP2 DM may have aggressive calcinosis and greater risk of malignancy [1, 4]. A patient with anti-SRP DM may have early-onset atrophy, myocardial involvement, and a delayed response to therapy, which is important to identify in planning management and patient expectations [5].

Identifying the specific signs associated with the different subtypes as well as the MSA/MAA involved is critical in the management of these patients and will impact on outcomes [4].

## 2. Case 1

**Figure 1:**
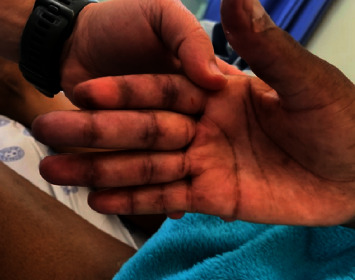
Inverse Gottron's papules in MDA 5 DM.

A 38-year-old African woman with HIV on anti-retroviral therapy presented with inflammatory arthritis and ulcerating lesions over the dorsal aspects of the MCP joints of both hands. Inverse Gottron's papules, commonly associated with anti-MDA 5 DM, were present (Figure 1). Her myositis antibody profile confirmed anti-MDA 5 MSA, and she was initiated on mycophenolate mofetil and methylprednisolone. Her HRCT revealed mild NSIP, and rituximab was added to her regimen. She is currently doing well on MMF and cyclosporin maintenance.

**Figure 2:**
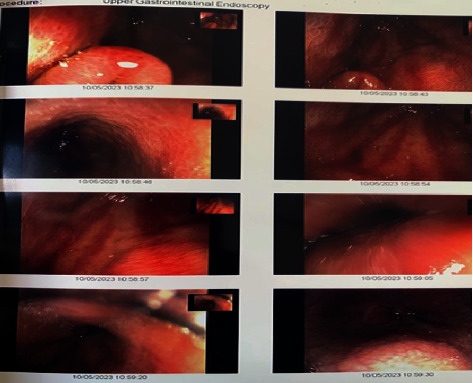
GAVE in MDA 5 DM.

A gastroscopy was done for dysphagia and revealed a watermelon stomach as depicted in Figure 2. Notably, no additional antibody was positive to suggest overlap with systemic sclerosis which is commonly associated with gastric antral vascular ectasia (GAVE). A review of the literature revealed no association of GAVE with MDA 5 DM. The proposed pathophysiology in GAVE is related to vascular phenomena. Abnormal nail fold capillaries, telangiectasia, and Raynaud's phenomenon are common to both systemic sclerosis and dermatomyositis which may explain the presence of GAVE in this patient. GAVE however is not frequently reported in dermatomyositis [6, 7].

## 3. Case 2

**Figure 3:**
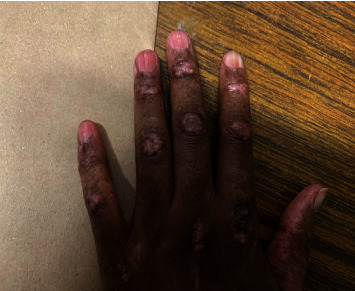
Hypopigmented Gottron's papules in TIF1y DM.

**Figure 4:**
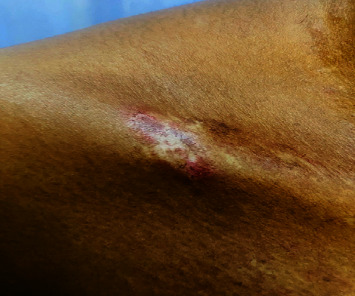
Red on white patch in TIF1y DM.

A 30-year-old African woman presented in 2020 with a heliotrope rash, facial photosensitive rash, shawl sign, v sign, truncal poikiloderma, sleeve sign, hypopigmented Gottron's papules (Figure 3), calcinosis, and a specific sign called red on white patch (Figure 4). Her myositis antibody profile was anti-TIF1y-positive in 2023 after the test became available. She was investigated for malignancy which revealed a markedly thickened gall bladder wall on CT scan, for which gallbladder cancer was suspected. The patient underwent resection and histology confirmed a benign lipoma and no malignant process.

The red on white patch detected in this patient is a distinct and valuable sign that should not be overlooked. It is a hypopigmented lesion with telangiectasia due to vasculopathy. Detection of these lesions should alert the clinician to thoroughly investigate the patient for a malignancy. In resource-limited settings where myositis profiles are not freely accessible, this characteristic clinical sign may prove vital to the workup of the patient [8, 9].

## 4. Case 3

**Figure 5:**
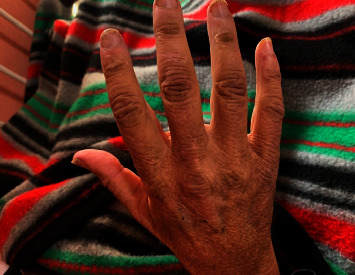
Acute Gottron's papules in SRP DM.

**Figure 6:**
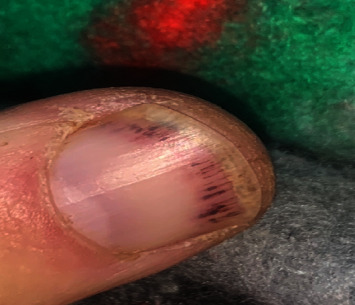
Splinter hemorrhages in SRP DM.

A 60-year-old Caucasian man presented with progressive muscle weakness and Raynaud's phenomenon. At presentation, he already had significant quadriceps atrophy and overt splinter hemorrhages (Figure 5). No skin rash was observed on admission; however, within a month of admission, he had developed Gottron's papules (Figure 6). Because of the atypical presentation, a muscle biopsy was done which revealed necrotizing polymyositis on histology. The myositis antibody profile confirmed an anti-SRP-positive result. The patient responded gradually on prednisone, IVIG, and azathioprine with normalization of CK with addition of low-dose methotrexate later. As expected, response to therapy and rehabilitation was slow, but at 4 months, the patient was walking unaided and has regained significant muscle mass. His echocardiogram revealed left ventricular hypertrophy in the absence of hypertension.

Skin involvement in anti-SRP myositis is seldom reported. Splinter hemorrhages are less frequently reported in dermatomyositis compared to nail fold capillary changes. In the literature, one study identified splinter hemorrhages as a potential sign of disease activity. In the patient described in this paper, the splinter hemorrhages did resolve congruent with response to therapy [10].

## 5. Summary

The clinician should be vigilant in detecting certain unique clinical signs such as inverse Gottron's papules in anti-MDA 5 DM, red on white patch in anti-TIF1y DM, and early-onset atrophy in anti-SRP DM. These clinical findings could result in earlier detection of specific subtypes of IIM and implementation of more targeted therapy with improved outcomes. In resource-limited settings where myositis antibody profiles are not freely available, clinical phenotyping is an important skill to possess in practice.
